# Non-fluorescent transient states of tyrosine as a basis for label-free protein conformation and interaction studies

**DOI:** 10.1038/s41598-024-57054-6

**Published:** 2024-03-18

**Authors:** Niusha Bagheri, Hongjian Chen, Mihailo Rabasovic, Jerker Widengren

**Affiliations:** 1 Experimental Biomolecular Physics, Department of Applied Physics, Royal Institute of Technology (KTH), Albanova University Center, 106 91 Stockholm, Sweden; 2 Laboratory for Biophysics, Institute of Physics Belgrade, Pregrevica 118, 11080 Zemun-Belgrade, Serbia

**Keywords:** Biological fluorescence, Molecular conformation, Fluorescence spectroscopy

## Abstract

The amino acids tryptophan, tyrosine, and phenylalanine have been extensively used for different label-free protein studies, based on the intensity, lifetime, wavelength and/or polarization of their emitted fluorescence. Similar to most fluorescent organic molecules, these amino acids can undergo transitions into dark meta-stable states, such as triplet and photo-radical states. On the one hand, these transitions limit the fluorescence signal, but they are also highly environment-sensitive and can offer an additional set of parameters, reflecting interactions, folding states, and immediate environments around the proteins. In this work, by analyzing the average intensity of tyrosine emission under different excitation modulations with the transient state monitoring (TRAST) technique, we explored the photo physics of tyrosine as a basis for such environment-sensitive readouts. From how the dark state transitions of tyrosine varied with excitation intensity and solvent conditions we first established a photophysical model for tyrosine. Next, we studied Calmodulin (containing two tyrosines), and how its conformation is changed upon calcium binding. From these TRAST experiments, performed with 280 nm time-modulated excitation, we show that tyrosine dark state transitions clearly change with the calmodulin conformation, and may thus represent a useful source of information for (label-free) analyses of protein conformations and interactions.

## Introduction

Fluorescence readouts are extensively used to characterize protein conformations and interactions. With extrinsic fluorophores, such studies can be performed with high specificity, single-molecule sensitivity and at high spatial and temporal resolution^1^. However, covalent labeling modifications may result in structural and functional changes in the proteins and the labeling can be tedious, time-consuming, and sometimes difficult to achieve in a site-selective manner^2^. To avoid these problems, the intrinsically fluorescent amino acid residues tryptophan (Trp), tyrosine (Tyr) and phenylalanine (Phe) can be used for label-free protein studies^3^. Due to its much stronger fluorescence intensity, Trp is far more used than Tyr or Phe^1^, and Tyr fluorescence in proteins is usually quenched in Trp-containing proteins. On the other hand, Tyr residues are typically far more abundant in proteins and peptides^4^. Several peptides and smaller proteins of large biomedical relevance, such as A-beta peptides and alfa-synuclein, do not contain any Trp residues, but one or several Tyr residues, and their fluorescence has been useful to study structural and aggregation properties of e.g. beta-amyloid peptides and their role in Alzheimer´s disease and possible disease inhibitors^5–8^. Given the limited fluorescence brightness of Tyr, it is in studies of such peptides and proteins motivated to extract all possible information content in the emitted fluorescence, and time-resolved lifetime measurements have proven to add useful information to spectrofluorometer measurements^4–9^. Yet, there is additional information to be considered. In this work, we investigated the blinking properties of Tyr, due to transitions to and from long-lived dark states, as a possible additional information source in intrinsic Tyr fluorescence studies of proteins and peptides.

The long lifetimes of excitation-induced, transient states of fluorophores in general, such as triplet, photo-ionized and -isomerized states, give them correspondingly more time to interact with their local surrounding and make these states potentially very environment-sensitive. The resulting blinking behavior of fluorophores can be studied in a straightforward manner by fluorescence correlation spectroscopy (FCS)^10,11^. However, such FCS measurements require single-molecule detection (SMD) conditions, bright and photostable fluorophores, and detection with high sensitivity and sub-microsecond time-resolution. As an alternative, transient state (TRAST) spectroscopy can be used, which analyses the time-averaged fluorescence intensity, $\langle {F}_{exc}\rangle ,$ detected from fluorophores subject to a modulated excitation. With the modulation systematically varied over the time range of the dark state transitions of the fluorophores, dark state transitions in fluorophores can be determined from how $\langle {F}_{exc}\rangle$ varies with the modulation characteristics^12–15^. In contrast to FCS, TRAST does not rely on SMD conditions, nor on a high time resolution in the detection. TRAST thus makes it possible to extend dark state transition studies to fluorophores in more demanding samples with limited signal-to-background conditions, and to fluorophores with lower brightness, including auto-fluorescent compounds, including tryptophan (Trp)^16^, NADH^17^, and flavin compounds^18^. Based on previous work, demonstrating the use of TRAST to monitor dark state transitions of Trp as a basis for label-free protein conformational studies^16^, we investigated in this work the prospects of using the blinking properties of Tyr, with an order of magnitude lower molecular brightness than Trp, for the same purpose. From our TRAST experiments, we first established a photophysical model describing the blinking of Tyr, which was dominated by photo-induced electron transfer. Then, based on this model, we could show how TRAST measurements based on Tyr fluorescence could be used to monitor conformational changes in a Tyr containing protein (Calmodulin) with no Trp residues.

## Methods and materials

### Transient state (TRAST) spectroscopy/imaging–basic concept and theory

**Figure 1:**
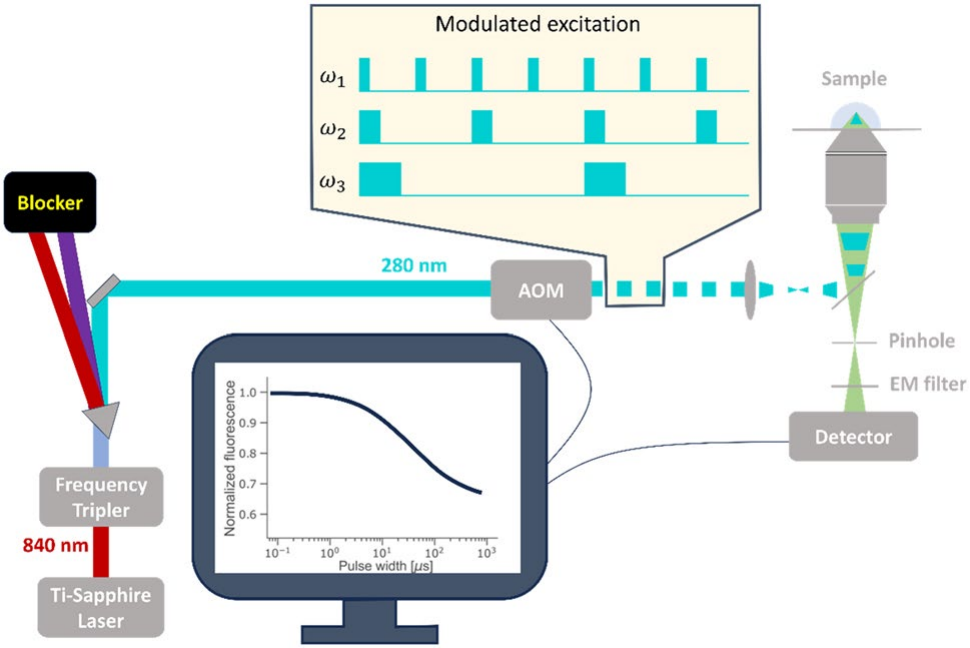
Schematic representation of the TRAST setup applied in this study, see main text for details.

In TRAST spectroscopy/imaging, the population dynamics of non-fluorescent, long-lived transient states of fluorescent molecules are determined from the average fluorescence intensity detected in the sample, when subject to different excitation pulse trains^12–15^ (Fig. 1).

For fluorescent molecules in a sample, subject to a low duty cycle rectangular excitation pulse train with *M* pulses of duration $w$, a time-averaged, normalized fluorescence intensity can be defined (see SI Section S1 for details):

1 $${\langle {F}_{exc}\left(w\right)\rangle }_{norm}=\frac{1}{M}\sum_{i=1}^{M}{\left(\frac{1}{w}\underset{0}{\overset{w}{\int }}S(t)dt\right)}_{i}=\frac{1}{w}\underset{0}{\overset{w}{\int }}S(t)dt$$

Here, *S*(*t*) represents the joint population probability of the fluorescent molecules to be in either the ground or excited singlet state (ready to undergo excitation-emission cycles), and 1-*S*(*t*) is correspondingly the fraction of photo-induced dark states, formed at time *t* after onset of a rectangular excitation pulse.

### Experimental setup and measurement procedure for TRAST

A home-built confocal microscope setup was used for the TRAST and FCS measurements, as previously described^16^. In short, a mode-locked Ti:Sapphire laser (Mira 900, Coherent, pumped by Nd:Vanadate laser (Verdi™ V-10, Coherent) operating at 840 nm, pulse width ~ 120 fs FWHM, 76 MHz repetition rate), frequency-tripled with a third harmonic generator (INRAD M/N 5-050, Inrad Inc.) to 280 nm was used as excitation source. An acousto-optic modulator (AOM;MQ200-B80A1-266,AA Opto-Electronics, Orsay Cedex) was used for intensity modulation. The beam was focused into the sample by a microscope objective (Ultrafluar 100/1.25 glyc, Zeiss). The confocal detection volume was determined by FCS measurements of 2-Aminopurine in aqueous solution (Fig. S1), yielding a $1/{e}^{2}$ radius in the focal plane of ~ 400 nm. Fluorescence from the sample was collected through the same objective, separated from excitation light by a dichroic beam splitter (FF310-Di01, Semrock, Inc.), focused onto a 75 µm diameter pinhole in the image plane and was then spectrally filtered (FF02-320/40 single-band bandpass filter, Semrock), then split by a polka dot beam splitter (50:50, Thorlabs), focused and finally detected by two single photon counting photomultiplier tubes (H7360-02, Hamamatsu). The signals were recorded with a PCI-6602 counter/timer card (National Instrument Crop) for subsequent TRAST analysis.

Upon application of square-wave excitation pulse trains in the setup described above, TRAST curves were generated by recording ${\langle F(w)\rangle }_{norm}$ for up to 30 different excitation pulse trains, with the pulse width *w* varied between 100 and 10 ms. The duty cycle ɳ was kept at 1% to ensure a complete relaxation of tyrosine to the ground singlet state in-between pulses. For the experiments in solution, the sample was enclosed between a plastic cap and the quartz cover slide to avoid any evaporation and concentration changes during the measurements.

### TRAST data analysis

Data analysis was performed using software implemented in MATLAB, and as previously described^15,16^. In short, the experimental $\langle {F}_{exc}\left(w\right)\rangle$data was pre-processed by subtracting detector dark counts. Drifts in sample concentration arising from bleaching were corrected for by repetitive reference measurements throughout each experiment, using a short pulse width, ${w}_{0}$, to avoid dark state buildup.

To fit the data to a chosen photophysical model, simulated TRAST curves were generated by calculating ${\langle {F}_{exc}\left(w\right)\rangle }_{norm}$ based on the model using Eq. (1) for each pulse width, *w*. The excitation rate, ${k}_{01}\left(\overline{r }\right)=\sigma {\Phi }_{exc}\left(\overline{r }\right)$, was estimated using an excitation cross section of $\sigma =4.47\cdot {10}^{-18}$ cm^2^^19^, and assuming a Gaussian distribution of the excitation photon flux ${\Phi }_{exc}\left(\overline{r }\right)$, with a focal beam radius (1/e^2^) of 400 nm, as determined from FCS measurements on 2-Aminopurine in water (see “Results” section). To increase computational speed, an average excitation rate within the detection volume was assumed^16^;

2 $${\overline{k} }_{01}=\frac{\int \sigma {\Phi }_{exc}(\overline{r })\overline{{S }_{1}}(\overline{r })CEF(\overline{r })dV}{\int \overline{{S }_{1}}(\overline{r })CEF(\overline{r })dV}$$

where $\overline{{S }_{1}}\left(\overline{r }\right)=\sigma {\Phi }_{exc}(\overline{r })/({k}_{10}+\sigma {\Phi }_{exc}\left(\overline{r }\right))$, i.e. the S_1_ population at onset of excitation, after equilibration between the singlet states, but before dark state build-up. $CEF\left(\overline{r }\right)$ represents the collection efficiency function of the confocal setup used (Fig. 1). From Eq. (1), a simplified expression for ${\langle {F}_{exc}\left(w\right)\rangle }_{norm}$ can then be obtained, given by

3 $${\langle {F}_{exc}\left(w\right)\rangle }_{norm}=\frac{1}{w}\underset{0}{\overset{w}{\int }}{\frac{{k}_{10}+{\overline{k} }_{01}}{{\overline{k} }_{01}}}S_{1}\left(t\right)dt$$

The remaining model parameters were then optimized using Eq. (3), and an iterative non-linear least square approach to match the calculated curves to the experimental data. Multiple TRAST curves could also be fitted simultaneously, with each rate being specified as global or independent between curves.

### Sample preparation

A stock solution of 160 µM of L-tyrosine in DPBS (ThermoFisher Scientific) was freshly prepared and further diluted to 20 µM for the TRAST measurements, with pH set to 7, or as stated in the text. All chemicals were purchased from Sigma Aldrich, if not stated otherwise. Deoxygenation experiments were performed in a sealed container, where the solution was bubbled with nitrogen gas for twenty minutes before measurements. During the experiment, a low flow of nitrogen was applied over the sample to avoid re-oxygenation.

Calmodulin (CaM, purified from bovine brain Lyophilized from 30 mM HEPES, 1 mM CaCl_2_, 0.1 mM DTT, pH 7.4), reconstituted to a stock concentration of 1.858 mg/mL, was subsequently diluted to 20 μM for measurements in 30 mM HEPES (pH 7.4). The 20 μM CaM solution was considered calcium-saturated (hereinafter denoted CaM-Ca^2+^), given that it was expected to contain approximately 180 μM Ca^2+^, as indicated by the provided lyophilization information from the supplier (Sigma Aldrich). To obtain a calcium-free calmodulin solution (hereinafter denoted CaM-w/o-Ca^2+^), an excess of EGTA (Ethyleneglycol- bis(β-aminoethyl)-N,N,Nʹ,Nʹ-tetraacetic Acid) was introduced to chelate all calcium ions, while approximately 200 μM of free EGTA remained in the solution.

## Results

### TRAST experiments on free Tyr, photophysical model and environmental effects

**Figure 2:**
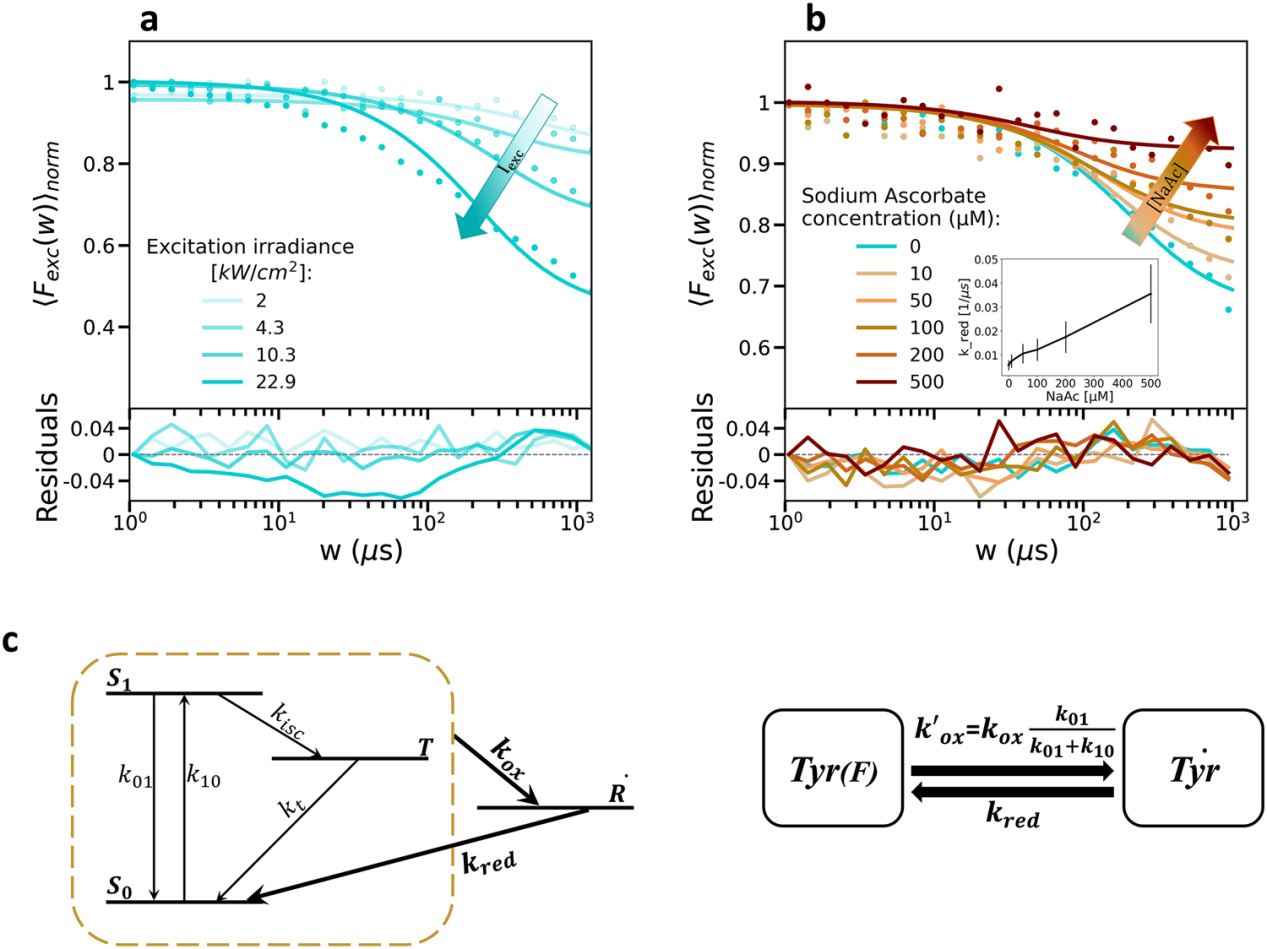
(**a**) TRAST curves recorded at different *I*_*exc*_ from 20 µM Tyr in water (dotted). Fitted TRAST curves (lines) generated as described in the main text, with residuals (bottom). Arrow indicates the increase of I_exc_ (from 2.0 to 22.9 kW/cm^2^). (**b**) TRAST curves recorded at *I*_*exc*_ = 10.3 kW/cm^2^ from the same type of Tyr sample, with [NaAc] ranging from 0 to 500 µM. The arrow indicates the increase of [NaAc]. Inset: Fitted *k*_*red*_ values vs [NaAc]. Error bars denote standard deviations. (**c**) Four-state photophysical model for Tyr, see main text for details. Based on this model, the *k*_*ox*_ and *k*_*red*_ rates (bold and enlarged in the figure) were determined to 19.2 ± 3.4 µs^-1^ (from Tables S1–S3, S5) and 0.0037 ± 0.0006 µs^-1^ (from Tables S1, S2 and S5), respectively. (**d**) Simplified two-state model used in the fitting of the experimental TRAST curves, see main text for details.

As a basis for the further experiments, we initially established a photophysical model for tyrosine, as observed in our TRAST experiments. As a major parameter influencing the dark state transitions, we first determined the excitation intensity, ${I}_{exc}$, applied onto the Tyr samples. For this, the 1/e^2^ radius of the laser beam in the focal plane after the objective was determined to 400 nm by fluorescence correlation spectroscopy (FCS) measurements of 2-Aminopurine in water (Supplementary, Fig. S1) and by TRAST experiments , using Tryptophan^16^ for calibration. With the beam dimensions determined, and the excitation intensities onto the samples then properly estimated, we next recorded TRAST curves of Tyr in PBS subject to different ${I}_{exc}$ (2–23 kW/cm^2^), as shown in Fig. 2a. In the set of TRAST curves, we observed an overall decay starting at around 100 µs and lasting up to the longest pulse durations (1000 µs). This decay is about two orders of magnitude slower than expected from a typical triplet state relaxation of a fluorophore in an air-saturated water solution^10,15^. Moreover, the decay rate of the T_1_ state of Tyr in air-saturated water has been reported to ${k}_{t}=$5.75 µs^−1^^20^, about an order of magnitude higher than for most fluorophores. This suggests that the triplet state build-up is minor, and that it would take place at a faster time scale than recorded in the TRAST experiments. TRAST curves recorded from corresponding deoxygenated Tyr samples did not show any significant differences, compared to curves recorded under air-saturated conditions (Supplementary Fig. S2). This suggests that in the absence of oxygen, a potent triplet state quencher, there are other major deactivation pathways present, keeping the T_1_ state population still very low.

To further investigate the dark state transition underlying the decay in the recorded TRAST curves (Fig. 2a), we added sodium ascorbate (NaAc) as a reducing agent. We then found that NaAc itself showed fluorescence under our experimental conditions (280 nm excitation), compared to the relatively weak Tyr fluorescence. By adding a band-pass emission filter (300–340 nm) the NaAc fluorescence could however be successfully suppressed. With NaAc added to the free Tyr in aqueous solution in different concentrations ([NaAc] = 0-500 µM, ${I}_{exc}$=10.3 kW/cm^2^), a significant decrease in the dark state relaxation amplitude was observed in the recorded TRAST curves (Fig. 2b). From previous flash photolysis studies of Tyr it has been inferred that a prominent radical state formation can take place from the lowest triplet, T_1_, state of Tyr^20^. The radical state may first be formed as a cation by photo-oxidation. However, this cation has been found to be very short-lived^21^ and mainly due to its low pK_a_ (~ − 2)^22^ to essentially only occur concomitant with proton transfer into a neutral radical, $\dot{{\text{R}}}$. We thus assign ${\dot{R}}$ as the dark state formed and prominent radical state formation (by photo-oxidation and concomitant proton transfer) from the T_1_ state can also explain the lack of a significant triplet state buildup in our TRAST measurements. The experimental findings can be housed within a four-state model for Tyr, including the S_0_, S_1_, T_1_ and $\dot{{\text{R}}}$ states (Fig. 2c). In this model, T_1_ population buildup via intersystem crossing (*k*_*isc*_) from S_1_ is low because of a prominent triplet decay rate (*k*_*t*_) to S_0_, alternatively, because the photo-oxidation rate (*k*_*ox*_) with concomitant proton transfer to $\dot{{\text{R}}}$ dominates over *k*_*t*_. Given that *k*_*ox*_ >  > *k*_*t*_, or that the singlet–triplet transitions take place on a much faster time scale than transitions to and from $\dot{{\text{R}}}$, we can reduce our model to a two-state model (Fig. 2d), including a fluorescent state (Tyr(F), within which the singlet–triplet transitions take place) and a photo-induced, non-fluorescent radical (${\dot{{\text{Tyr}}}})$ state. We can then assign an effective photo-oxidation rate from Tyr(F) with concomitant proton transfer to $\dot{{\text{Tyr}}}$ as:

4 $$k_{{ox}}^{{\prime }} = k_{{ox}} \frac{{k_{{01}} }}{{k_{{01}} + k_{{10}} }}$$

and a not excitation-driven reduction rate, *k*_*red*_, from $\dot{{\text{Tyr}}}$ back to Tyr(F).

Based on the model in Fig. 2d, TRAST curves were numerically calculated and fitted to the experimental TRAST curves as described in “Methods and materials”, with *k*_*ox*_ fitted globally and *k*_*red*_ fitted individually to the TRAST curves (Fig. 2a, b). Fitted parameter values are reported in the Supplementary Tables S1–S5. In Fig. 2a, the fitted curves could well reproduce the experimental data, except for the TRAST curve recorded at the highest *I*_*exc*_ (22.9 kW/cm^2^), for which there is a deviation from the fitted curve in the 10–100 µs time range. This is at least one order of magnitude slower than what can be expected for a triplet state relaxation^10,16^. Likewise, rotamer transitions can take place in Tyr, but typically occur in the nanosecond time scale^9^. A possible contribution to this deviation is rather diffusion recovery of Tyr(F) into the confocal detection volume, which can be expected to occur at higher *I*_*exc*_ and when $\omega$ approaches the diffusion time of Tyr through the detection volume, i.e. in the same time range as the observed deviation (as inferred from the FCS experiments of 2-AP, Fig. S1). As previously shown by TRAST simulations^23^, this effect can lead to under-estimation of transition rates to dark-states and over-estimation of dark state recovery rates. To minimize such effects, we did not perform TRAST experiments beyond this *I*_*exc*_ level (22.9 kW/cm^2^). For the TRAST curves in Fig. 2b, fitting of *k*_*ox*_ (globally fitted) and *k*_*red*_ (individually fitted to each curve) could well reproduce the experimental data, with the fitted *k*_*red*_ values showing a linear dependence to [NaAc], with a slope of ${k}_{Qred}=$ 5.8 × 10^7^ M^−1^ s^−1^. This effect is well in line with the expected effect of a reducing agent on a cation radical state, as observed for e.g. rhodamine fluorophores, but the slope is about an order of magnitude lower for tyrosine, which may be attributed to the likely neutral charge of the radical state in this case. All fitted parameter values from the TRAST curves in Fig. 2a, b are listed in Tables S1 and S3.

Next, we studied effects of potassium iodide (KI) on the dark state kinetics of Tyr. KI is well known to enhance *k*_*isc*_ by a I^-^ heavy atom effect, but has also been found to enhance k_t_ by means of electron transfer for dyes with excitation maximum in the blue-green range^24^. Moreover, KI can also act as a reducing agent, contributing to the recovery of photo-induced radicals of the fluorophores back into their fluorescent states^24^. For Tyr in aqueous solution addition of KI in different concentrations ([KI] = 0–171 mM) no evidence of triplet state population/relaxation on TRAST curves was observed (Fig. S3, with fitted parameter values in table S4). However, with increasing [KI], a clear reduction of the dark state relaxation amplitude could be noted. Similar to NaAc, this reduction is consistent with the role of KI as a mild reductant^24^ and supports the assignment of the decay in the TRAST curves to a photo-induced radical state formation.

As a final test on free Tyr, we studied if a change of the solvent, from aqueous solution (H_2_O) to heavy water (D_2_O), had any effects on the dark state kinetics. Use of D_2_O instead of H_2_O shifts the major Raman band of the solvent to shorter wavelengths, offering a possibility to reduce its overlap with the fluorescence emission band. Use of D_2_O has also been found to reduce non-radiative excited state decay rates in near-IR cyanine fluorophores, mediated by hydrogen-bond-assisted contributions and resulting in higher fluorescence yields^25^. For Tyr in D_2_O, a slightly (20–30%) higher fluorescence intensity could be recorded, compared to in H_2_O (inset, Fig. S4), while in TRAST curves recorded from Tyr in D_2_O at different *I*_*exc*_ (Fig. S4) no specific differences were observed compared to corresponding H_2_O measurements (Fig. S4, with fitted rate parameters in Table S2). This suggests that hydrogen-bond interactions are not affecting the dark state kinetics of Tyr, and that D_2_O measurements can offer a strategy to reduce contributions from Raman scattering and increase signal-to-background conditions.

Overall, from the TRAST measurements of free Tyr we find some variation in the determined *k*_*ox*_ and *k*_*red*_ rates for conditions under which no changes in these rates are expected (*k*_*ox*_ values listed in Tables S1–S3, S5 and *k*_*red*_ values listed in Tables S1, S2, S5). The determined rates are however relatively consistent with each other, and from these rates we calculated the following average rates and uncertainties: *k*_*ox*_ = 19.2 ± 3.4 µs^−1^, *k*_*red*_ = 0.0037 ± 0.0006 µs^−1^.

### TRAST experiments on Calmodulin (CaM)

**Figure 3:**
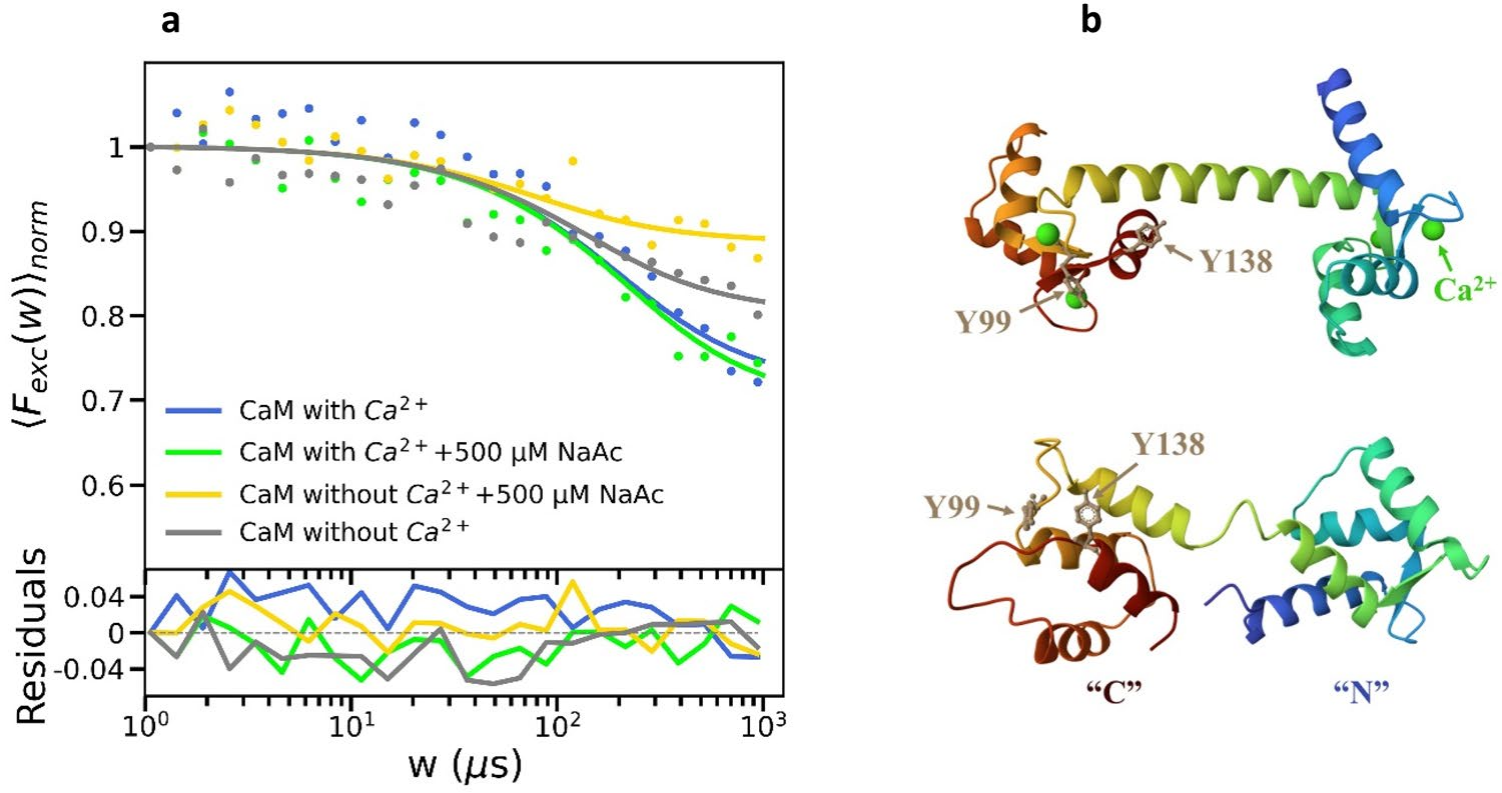
(**a**) Experimental TRAST curves recorded from 20 µM CaM in 30 mM HEPES, pH 7.4 (dotted), and fitted TRAST curves (lines) generated as described in the main text, with residuals (bottom). The TRAST curves were measured with, or without Ca^2+^, and with or without 500 µM NaAc. (**b**) Ribbon diagram of calcium-free and calcium-saturated CaM, as determined by X-ray diffraction^26^ and NMR spectroscopy^27^, respectively. Tyr residues are denoted by Y99 and Y138, and Ca2 + ions are denoted by green dots.

With a basic photophysical model for Tyr, as reflected in the TRAST experiments, with $\dot{{\text{Tyr}}}$ as the major dark state formed upon excitation, and with a distinct recovery from $\dot{{\text{Tyr}}}$ back to Tyr(F) promoted by adding a reductant (NaAc), we next explored if this dark state transition could be exploited as a readout of protein conformation states or interactions. As a test protein, we studied Calmodulin (CaM), which contains 148 amino acids, no Trp but two Tyr residues (Y99 and Y138). Upon binding of four Ca^2+^ ions, CaM changes its conformation (Fig. 3b) and a hydrophobic domain is formed^28^, which serves as an interface for interactions with various proteins and further transmission of the calcium signal. In label-free TRAST protein measurements based on Trp fluorescence^16^, differences in triplet state buildup in Trp could be used as a conformational state readout, reflecting different accessibilities of dissolved molecular oxygen to the Trp residue(s) of the protein. For Tyr, we did not observe any significant triplet state buildup in our TRAST experiments (Fig. 2a, b). However, instead of local accessibility to molecular oxygen, we could exploit the quenching of the $\dot{{\text{Tyr}}}$ state of the Tyr residues in CaM by NaAc, determined by the local accessibility of NaAc to these residues. Consequently, we monitored the effect of NaAc on CaM by TRAST experiments, for the two conformations of CaM; in the presence (Halo form, CaM-Ca^2+^) and absence (Apo form, CaM-w/o-Ca^2+^) of Ca^2+^. As seen in Fig. 3a, for CaM-Ca^2+^, no significant effect was seen in the TRAST curves upon adding 500 µM of NaAc. This indicates that Y99 and Y138 residues are shielded, with no or very limited access of NaAc to these residues. In contrast, without Ca^2+^ in the CaM sample, and CaM adopting an Apo conformation, adding NaAc resulted in a significant reduction of the $\dot{{\text{Tyr}}}$ population, reflecting that for CaM-w/o-Ca^2+^, the Tyr residues are more exposed to the solvent and accessible for NaAc. It can be noted that the $\dot{{\text{Tyr}}}$ population also was lower for CaM-w/o-Ca^2+^ than for CaM-Ca^2+^, which likely reflects that apart from the accessibility of NaAc, also the local environment of the Tyr residues can affect the $\dot{{\text{Tyr}}}$ population. For both forms of CaM, the recorded TRAST curves could be well fitted to the same model as for free Tyr, with fitted rate parameters given in Table S6.

## Discussion

Using Tyr fluorescence allows for label-free measurements on proteins without Trp. Apart from regular fluorescence parameters, monitoring of blinking due to dark state transitions within fluorophores can offer a complementary, rich source of information reflecting conformations and interactions of such proteins. However, the relatively weak fluorescence of aromatic amino acids makes such label-free blinking measurements on proteins challenging, and practically impossible by FCS or other single-molecule measurements. The TRAST technique offers a robust, more widely applicable technique to monitor fluorophore blinking, and with TRAST based on Trp fluorescence label-free protein conformation studies are possible^16^. Similarly, TRAST measurements on autofluorescent NADH^17^ and flavins^18^ have been demonstrated. In this study, we employed the TRAST technique and show that dark state transitions can be monitored also in Tyr, despite its order of magnitude lower brightness compared to Trp. Despite the more challenging signal-to-background conditions, we could measure dark state transitions of Tyr, establish a simple electronic state model dominated by photo-induced radical state formation, and show that the radical state formation can be reversed by adding a reductant in sub-mM concentrations. In CaM, with two Tyr residues, we then show how the accessibility of a reductant in the surrounding aqueous solution, i.e. the surface exposure of these residues in the protein, is reflected in the dark state transitions of the Tyr residues. This makes it possible to distinguish a closed from an open form of CaM and given the long (100 µs time range) lifetimes of the photo-induced radical state, also quite low-frequency collisional encounters between the Tyr residues and a reductant/quencher can be detected. This is in contrast to regular dynamic quenchers of Tyr fluorescence, for which collisions need to occur within the excited state lifetime of Tyr, on a nanosecond time scale. This longer time-window for collisional encounters/quenching can add sensitivity and can offer additional orthogonal information to regular fluorescence quenching experiments. While our results indicate that dark state transitions even in very weakly emitting species such as Tyr can be monitored and provide a basis for protein conformation and interaction studies, signal-to-background conditions still need to be considered. UV excitation may excite other species in the samples than Tyr. In our work, excitation of NaAc and its subsequent fluorescence emission was significant compared to the Tyr fluorescence but could be spectrally separated by adding an extra emission filter. As shown here, use of D_2_O instead of H_2_O can increase the fluorescence signal and allow better spectral separation of solvent Raman scattering from the Tyr fluorescence. In this work, we used a Ti:Sapphire laser and frequency tripler to tune the excitation wavelength. However, for future work, a UV diode laser can offer more simple, inexpensive turn-key alternatives for excitation, with the laser either directly modulated, or as in this work, with the laser emission modulated by an AOM. In recent works^29^, plasmonic resonance enhancement of Trp fluorescence has proven useful for label-free protein studies. Such approaches may also find use in combination with TRAST in blinking readouts, for protein conformational and interaction studies, based on an enhanced Trp as well as Tyr fluorescence signal, without requiring single molecule detection conditions.

### Supplementary Information

Supplementary Information.

## Data Availability

The experimental raw data behind this study has been placed at the Zenodo repository (10.5281/zenodo.10419469) for open access.
