# Supplementary material for: Non-fluorescent transient states of tyrosine as a basis for label-free protein conformation and interaction studies
Source: Sci Rep. 2024 Mar 18;14:6464. doi: 10.1038/s41598-024-57054-6 (PMC10948778; doi:10.1038/s41598-024-57054-6)
Supplement: Supplementary file 1 — Supplementary Information. [file 41598_2024_57054_MOESM1_ESM.docx]

**Supplementary Information**

**Non-fluorescent transient states of tyrosine as a basis for label-free protein conformation and interaction studies**

**Niusha Bagheri ^1^, Hongjian Chen ^1^, Mihailo Rabasovic ^2^, Jerker Widengren ^1,*^**

^1^ Royal Institute of Technology (KTH), Experimental Biomolecular Physics, Dept. Applied Physics, Albanova University Center 106 91 Stockholm, Sweden

^2^ Laboratory for Biophysics, Institute of Physics Belgrade, Pregrevica 118
11080 Zemun-Belgrade, Serbia

* Corresponding author (jwideng@kth.se)

### Section S1: Transient state (TRAST) spectroscopy/imaging – basic concept and theory

For a fluorescent molecule in a confocal detection volume, subject to a rectangular excitation pulse initiated at $t=0$, the resulting detected fluorescence intensity can be expressed:

$$\begin{aligned} F(t)=cq_{f}q_{D}\iiint\frac{k_{01}\left( \bar{r} \right)}{k_{01}\left( \bar{r} \right)+k_{10}}S\left( \bar{r},t \right) CEF\left( \bar{r} \right) dV\#(S1) \end{aligned}$$

where $\boldsymbol{c}$ is the concentration of the fluorescent molecule, $\boldsymbol{q}_{\boldsymbol{f}}$ is the fluorescence quantum yield, $\boldsymbol{q}_{\boldsymbol{D}}$ the overall detection quantum yield of the microscope, $\boldsymbol{CEF}\left( \bar{\boldsymbol{r}} \right)$ represents the collection efficiency function and $\boldsymbol{S}\left( \bar{\boldsymbol{r}}\mathbf{,}\boldsymbol{t} \right)$ the probability that a fluorophore, residing at position $\bar{\boldsymbol{r}}$ in the detection volume, is in either its ground (S_0_) or excited (S_1_) singlet state at time *t*. $\boldsymbol{k}_{\mathbf{10}}$ denotes the decay rate within the fluorophores from S_1_ to S_0_, $\boldsymbol{k}_{\mathbf{01}}\boldsymbol{=}\boldsymbol{\sigma}_{\boldsymbol{exc}}\boldsymbol{\Phi}_{\boldsymbol{exc}}\left( \bar{\boldsymbol{r}} \right)\boldsymbol{=}\boldsymbol{\sigma}_{\boldsymbol{exc}}{\boldsymbol{I}_{\boldsymbol{exc}}}/\boldsymbol{hv}$ represents the excitation rate from $\mathbf{S}_{\mathbf{0}}$ to $\mathbf{S}_{\mathbf{1}}$, where $\boldsymbol{\sigma}_{\boldsymbol{exc}}$ is the excitation cross section of the fluorophore, $\boldsymbol{\Phi}_{\boldsymbol{exc}}\left( \bar{\boldsymbol{r}} \right)$ the local excitation flux, $\boldsymbol{I}_{\boldsymbol{exc}}$ the excitation intensity and $\boldsymbol{hv}$ is the excitation photon energy ^18^. With a constant excitation starting at *t* = 0, the probability for a fluorophore to be in a singlet state (S_1_ or S_0_) at time t can in a general form be described by:

$$S(t)=1-\sum_{i=1}^{p} \left[ A_{i}-A_{i}e^{-\lambda_{i}t} \right] (S2)$$

Here, *λ_i_* are the eigenvalues, i.e. the rates of relaxation modes of *S(t)* upon onset of constant excitation, and *A_i_* the related amplitudes, reflecting the population build-up of the different photo-induced non-fluorescent states at steady-state ($t\gg1/\lambda_{i})$. With a suitable initial condition, typically *S(0)=*1. The relaxation process of $S(t)$ due to this dark state build-up can also be reflected in the time-averaged fluorescence signal generated by a rectangular pulse with a duration of $w$:

$$\begin{aligned} \left\langle F_{exc}(w) \right\rangle=\frac{1}{w}\int_{0}^{w} F(t)dt\#(S3) \end{aligned}$$

Here, the change of $\left\langle F_{exc}(w) \right\rangle$ with $w$ allows the population kinetics of long-lived, dark transient states, to be determined, which is the general basis for TRAST method. So-called TRAST curve are then generated by collecting $\left\langle F_{exc}(w) \right\rangle$ over multiple pulses, M, for different pulse durations, $w$, normalized by $\left\langle F_{exc}(w) \right\rangle$ recorded with a pulse train with short pulse duration, $w_{0}$:

$$\begin{aligned} \left\langle F_{exc}\left( w \right) \right\rangle_{norm}=\left( \frac{1}{M}\sum_{i=1}^{M} \left\langle F_{exc}\left( w \right) \right\rangle_{i} \right)/\left( \frac{1}{M_{0}}\sum_{i=1}^{M_{0}} \left\langle F_{exc}\left( w_{0} \right) \right\rangle_{i} \right)\#(S4) \end{aligned}$$

Here, $w_{0}$ is selected to be short enough to avoid the build-up of dark transient states ($w_{0}\ll1/\lambda_{i})$, yet longer than the anti-bunching relaxation time ^28^. By this normalization, the terms, $c$, $q_{D}$ and $q_{f}$ (S1) cancel out. With low duty cycles of the excitation pulse trains, so that the dark transient states of the fluorescent molecules can relax back to the singlet ground state, $S_{0}$, before the onset of next excitation pulse, all generated pulses in the pulse train can be treated as identical. We can then write:

Time-averaged fluorescence signal generated by a rectangular pulse with a duration of w was calculated and driven out in our previous publication^16^ as:

$$\left\langle F_{exc}\left( w \right) \right\rangle_{norm}=\frac{1}{M}\sum_{i=1}^{M} \left( \frac{1}{w}\int_{0}^{w} S(t)dt \right)_{i}=\frac{1}{w}\int_{0}^{w} S(t)dt (1)$$

Which M is number of pulses for different pulse duration, w. And S(t) is the relaxation from singlet states (S_0_ and S_1_) during the time.

**Section S2: *Supplementary FCS and TRAST curves and tabulated fitted rate parameters***


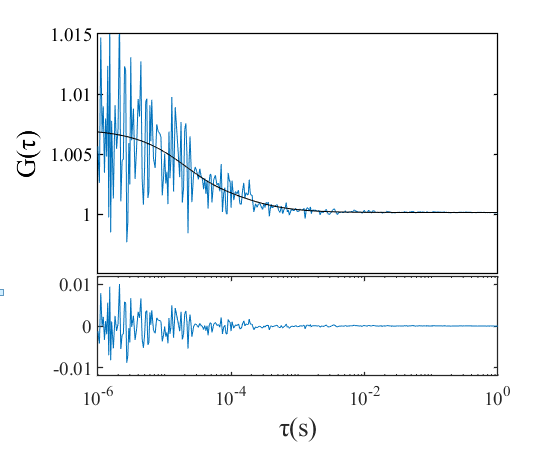


Figure S1. Fluorescence correlation spectroscopy (FCS) curve recorded from 2-Aminopurine (2-AP) in water, using the same setup as used for the TRAST experiments and with the same excitation beam dimensions. Based on the correlation time fitted to the FCS curve (τ_D_= 80µs) and with the diffusion coefficient of 2-AP given by D=4.4×10^-6^ cm^2^/s, the laser beam radius in the focal plane was estimated to ω=0.4µm. using a one component, diffusion only model).


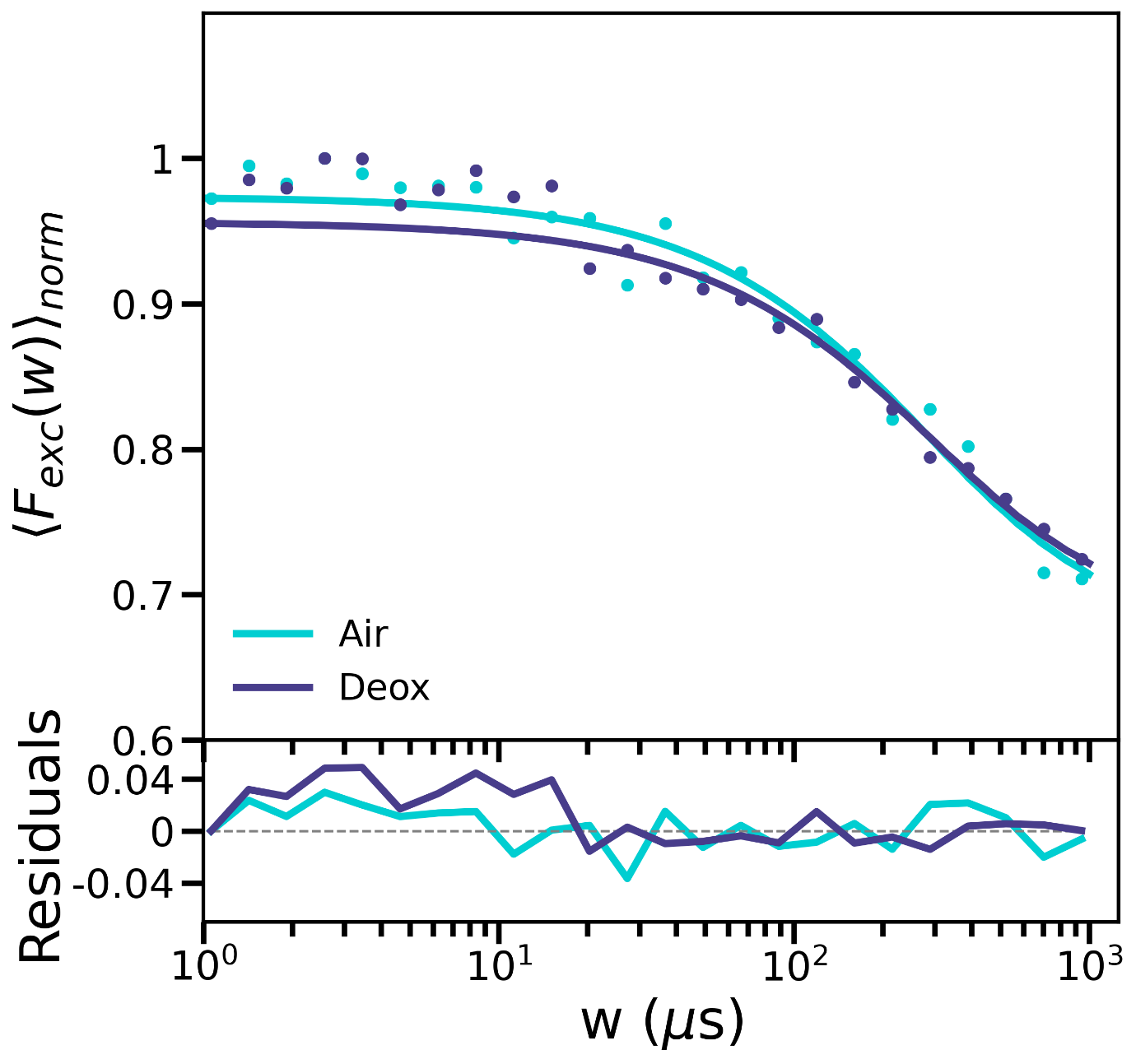


Figure S2. Normalized TRAST curves recorded from 20 µM Tyr in air-saturated (blue) and de-oxygenated (purple) water solution (dotted). Fitted TRAST curves (lines) generated as described in the main text, with residuals (bottom).


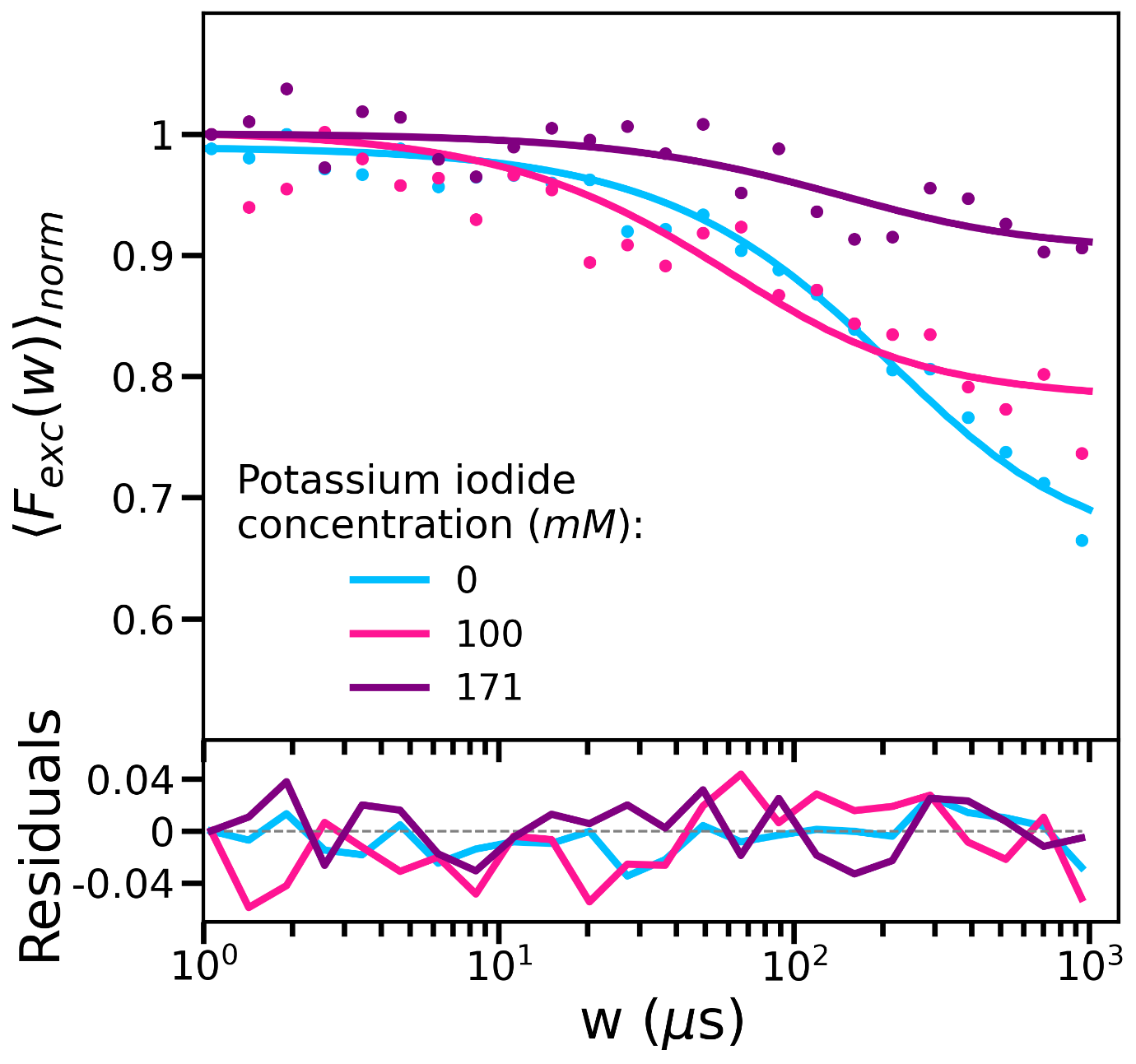


Figure S3. TRAST curves recorded in aqueous solution with different concentrations of potassium iodide (KI) added. Average excitation intensity 10.3 kW/cm^2^.


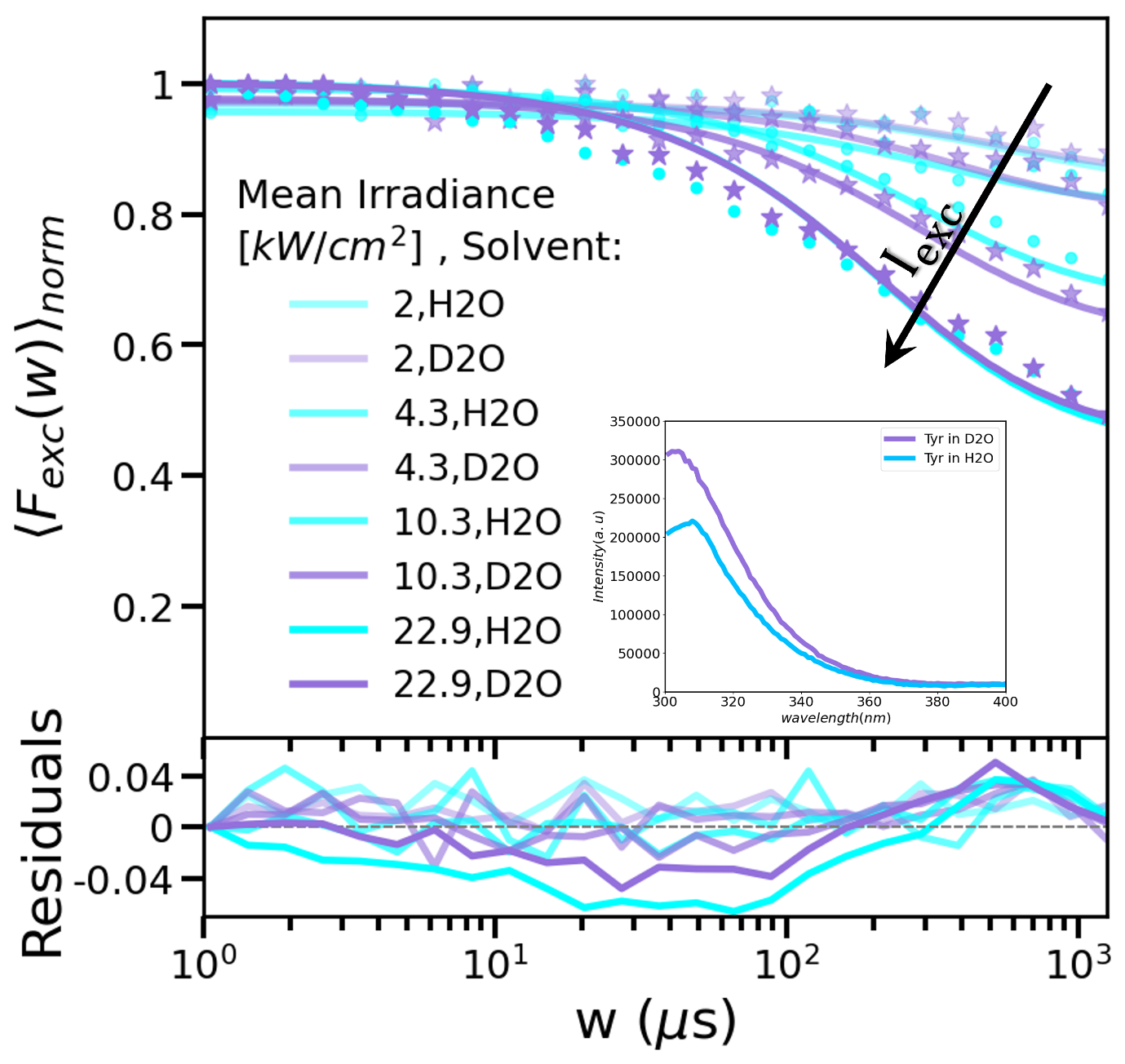


Figure S4. Normalized TRAST curves recorded from 20 µM Tyr in water (dotted) and heavy water (stars). Fitted TRAST curves (lines) were generated as described in the main text, with residuals (bottom). a) Average of 2.0, 4.3, 10.3, 22.9 kW/cm^2^. The arrow indicates the increase of I_exc_. Inset: Fluorescence spectra of Tyr (20 µM, 280 nm excitation) in water and heavy water.

**Supplementary Tables S1-S6. Fitted parameter values for tyrosine.**

| $I_{exc}[{kW}/{{cm}^{2}}]$ | *k_ox_*$[{\mu s}^{-1}]$ | $k_{red}[{\mu s}^{-1}]$ |
| --- | --- | --- |
| 2 | 16.1(global) | 0.0026 |
| 4.3 |  | 0.0046 |
| 10.3 |  | 0.0040 |
| 22.9 |  | 0.0034 |

Table S1. Fitted values for *k_ox_* (globally) and $k_{red}$ (individually) from TRAST curves of **Tyr in water**, recorded under **different excitation irradiances**, lifetime=3.6ns(fixed), (plotted in Figures 2a and S4).

| $I_{exc}[{kW}/{{cm}^{2}}]$ | *k_ox_*$[{\mu s}^{-1}]$ | $k_{red}[{\mu s}^{-1}]$ |
| --- | --- | --- |
| 2 | 16.1(global) | 0.0028 |
| 4.3 |  | 0.0038 |
| 10.3 |  | 0.0037 |
| 22.9 |  | 0.0036 |

Table S2. Fitted values for *k_ox_* (globally) and $k_{red}$ (individually) from TRAST curves of **Tyr in heavy water**, recorded under **different excitation irradiances**, lifetime=3.6ns (fixed), (plotted in Figure S4).

| $NaAc[\mu M]$ | *k_ox_*$[{\mu s}^{-1}]$ | $k_{red}[{\mu s}^{-1}]$ |
| --- | --- | --- |
| 0 | 21.6(global) | 0.0056 |
| 10 |  | 0.074 |
| 50 |  | 0.010 |
| 100 |  | 0.0121 |
| 200 |  | 0.0174 |
| 500 |  | 0.0355 |

Table S3. Fitted values for *k_ox_* (globally) and $k_{red}$ (individually) from TRAST curves of **Tyr in water**, recorded in different **sodium ascorbate concentrations,** I_exc_=10.3 kW/cm^2^, lifetime=3.6ns (fixed), (plotted in Figure 2b).

| $KI[mM]$ | *k_ox_*$[{\mu s}^{-1}]$ | $k_{red}[{\mu s}^{-1}]$ |
| --- | --- | --- |
| 0 | 20.1 | 0.0053 |
| 0.5 | 18.9 | 0.0059 |
| 20 | 44.1 | 0.018 |
| 100 | 46.1 | 0.0225 |
| 171 | 8.39 | 0.0108 |

Table S4. Fitted values for *k_ox_* and $k_{red}$ (both individually) from TRAST curves of **Tyr in water**, recorded in different **potassium iodide concentrations,** I_exc_=10.3 kW/cm^2^, lifetime=3.6ns (fixed), (plotted in Figure S3).

| O_2_ | *k_ox_*$[{\mu s}^{-1}]$ | $k_{red}[{\mu s}^{-1}]$ |
| --- | --- | --- |
| Air | 25.9 | 0.0043 |
| 60 min Deox | 23.2 |  |

Table S5. Fitted values for *k_ox_* (individually) and $k_{red}$ (globally) from TRAST curves of **Tyr in water**, **under air saturated and deoxygenated conditions,** I_exc_=10.3 kW/cm^2^, lifetime=3.6ns (fixed), (plotted in Figure S2).

| CaM environmental condition | *k_ox_*$[{\mu s}^{-1}]$ | $k_{red}[{\mu s}^{-1}]$ |
| --- | --- | --- |
| CaM with Ca^2+^ | 16.4(global) | 0.0062 |
| CaM with Ca^2+^ with NaAc |  | 0.0056 |
| CaM without Ca^2+^ with NaAc |  | 0.0194 |
| CaM without Ca^2+^ |  | 0.01 |

Table S6. Fitted parameter values for *k_ox_* (globally) and $k_{red}$ (individually) from TRAST curves of **Calmodulin with and without Ca^2+^ and NaAc**, lifetime=3.6ns (fixed), (plotted in Figure 3a).
